# Supplementary figures and images for: CRISPR-Cas is beneficial in plasmid competition, but limited by competitor toxin–antitoxin activity when horizontally transferred
Source: PLoS Biol. 2026 Feb 19;24(2):e3003658. doi: 10.1371/journal.pbio.3003658 (PMC12945316; doi:10.1371/journal.pbio.3003658)

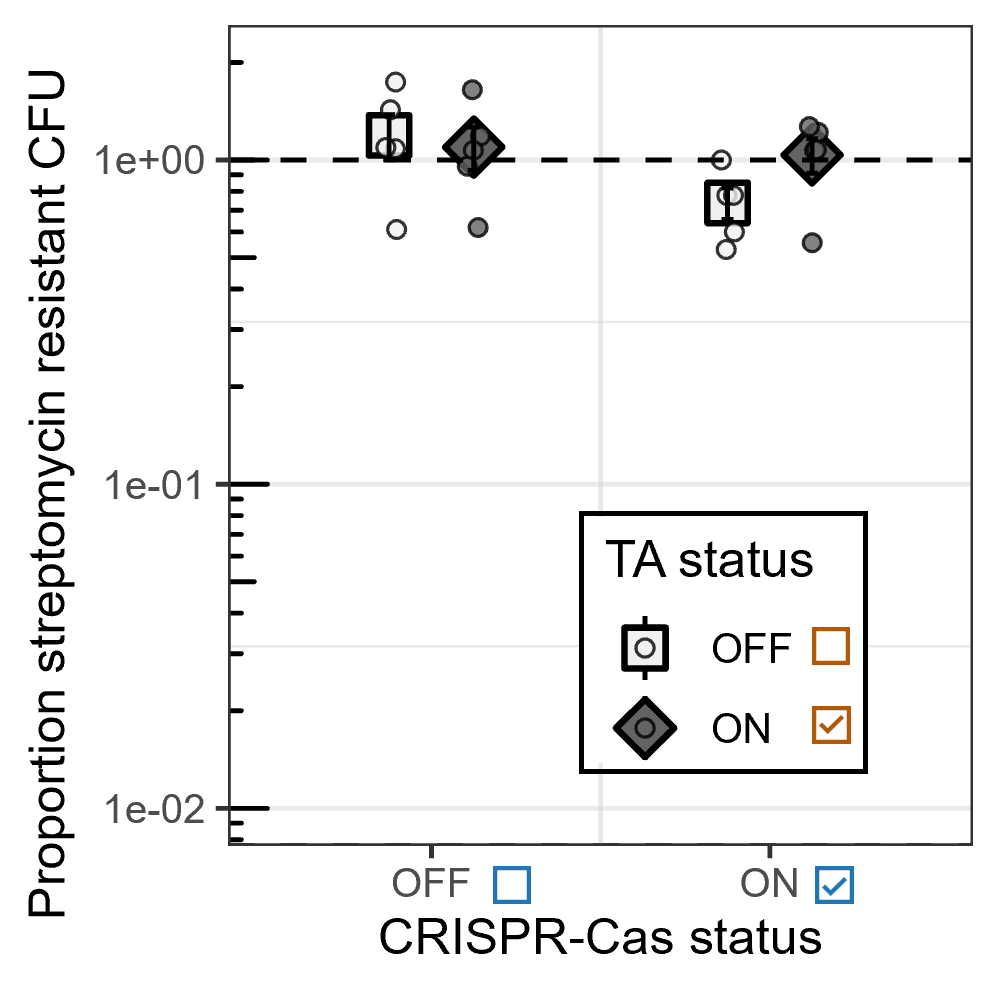

Supplement: S1 Fig — Mean ± standard error of the proportion of Streptomycin resistant Colony Forming Units (CFU) after mating was never significantly different from 1 (T test followed by Bonferroni adjustment for multiple testing; Table 7). Additionally, when stamp plating, only two individual clones out of 2,574 were found to be streptomycin sensitive, one of which was from a single-host control treatment. Vector presence was confirmed by PCR in all tested DH5α::SmR colonies. The data underlying this Figure can be found in S1 Data; the competitive outcome for this experiment is presented in S2 Fig. (TIF) [file pbio.3003658.s001.tif]

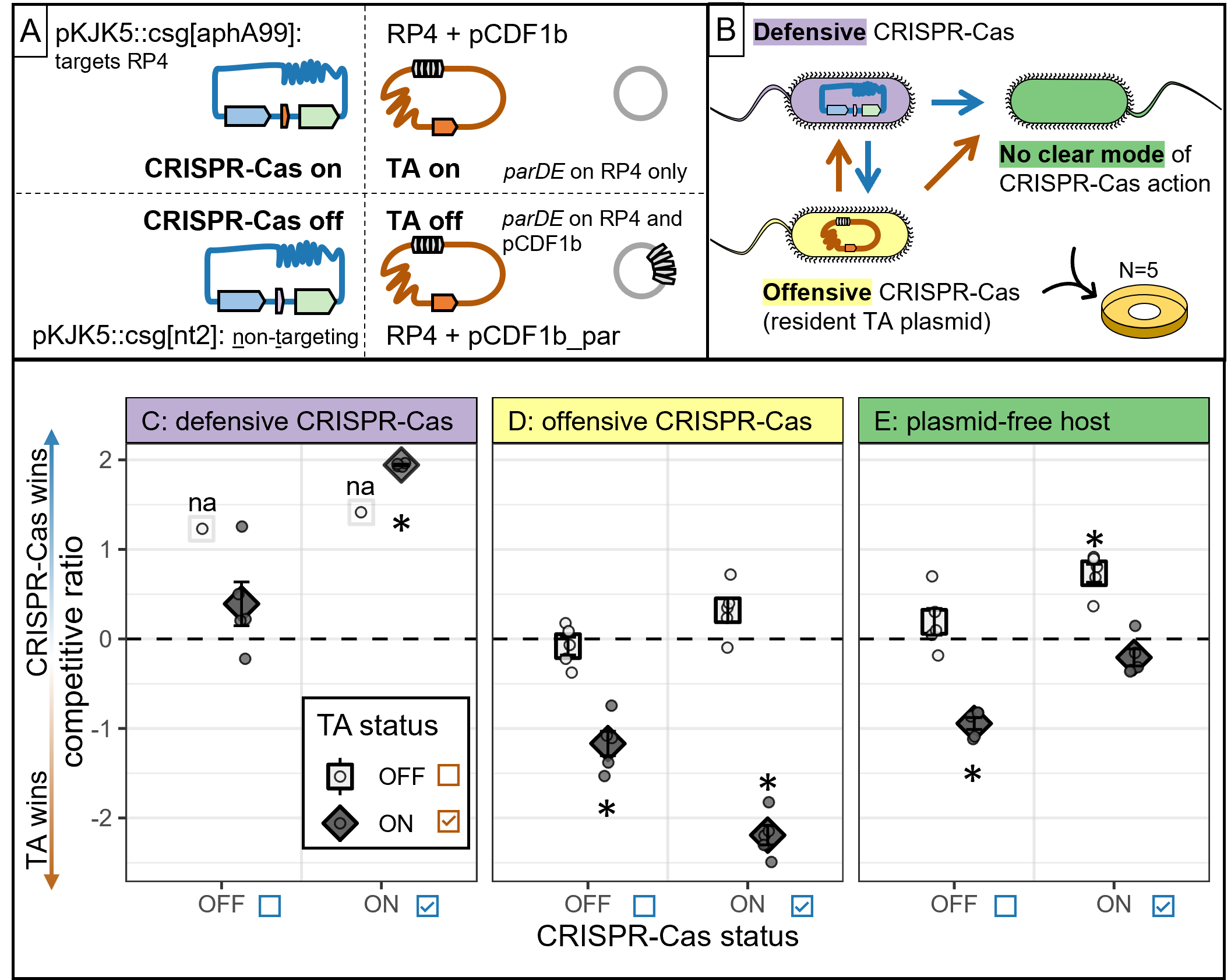

Supplement: S2 Fig — (A) CRISPR-Cas and TA activity were tweaked on competitor plasmids in a binary manner by switching each system on or off. pKJK5::csg carries a gene cassette encompassing cas9, sgRNA, GFP with either an RP4-targeting or non-targeting guide, RP4 naturally carries TA system parABCDE. (B) Plasmids were competed using three Escherichia coli DH5α hosts with different plasmid content, allowing us to assess competitive outcome under defensive, offensive, or no clear mode of CRISPR-Cas action. After filter mating, plasmid content of each host was assessed by selective plating. (C–E) Mean ± standard error of the competitive ratio (log odds ratio of pKJK5-carrying hosts/RP4-carrying hosts) describes outcome of plasmid competition (N = 5). Values >0 indicate CRISPR-Cas plasmid pKJK5 winning the competition, and values <0 indicate TA plasmid RP4 winning the competition for a certain host. The dashed line indicates a neutral outcome with competitive ratio = 0. Data are presented for treatments in which CRISPR-Cas and TA activity were toggled on or off in all combinations. Opacity of datapoints indicate number of replicates used to calculate means, this affects panel C TA-off data (N = 1) and panel C TA-on and CRISPR-Cas-on data (N = 4). Stars indicate significant differences from 0 as assessed by T test and Bonferroni adjustment for multiple testing; *p < 0.005 with α = 0.005; see Table 4 for all p values. na -not assessed due to N = 1 after removal of missing values (no carriage of TA plasmid RP4 recorded in 4 out of 5 replicates). The data underlying this Figure can be found in S1 Data. See S1 Text for additional information. (TIF) [file pbio.3003658.s002.tif]

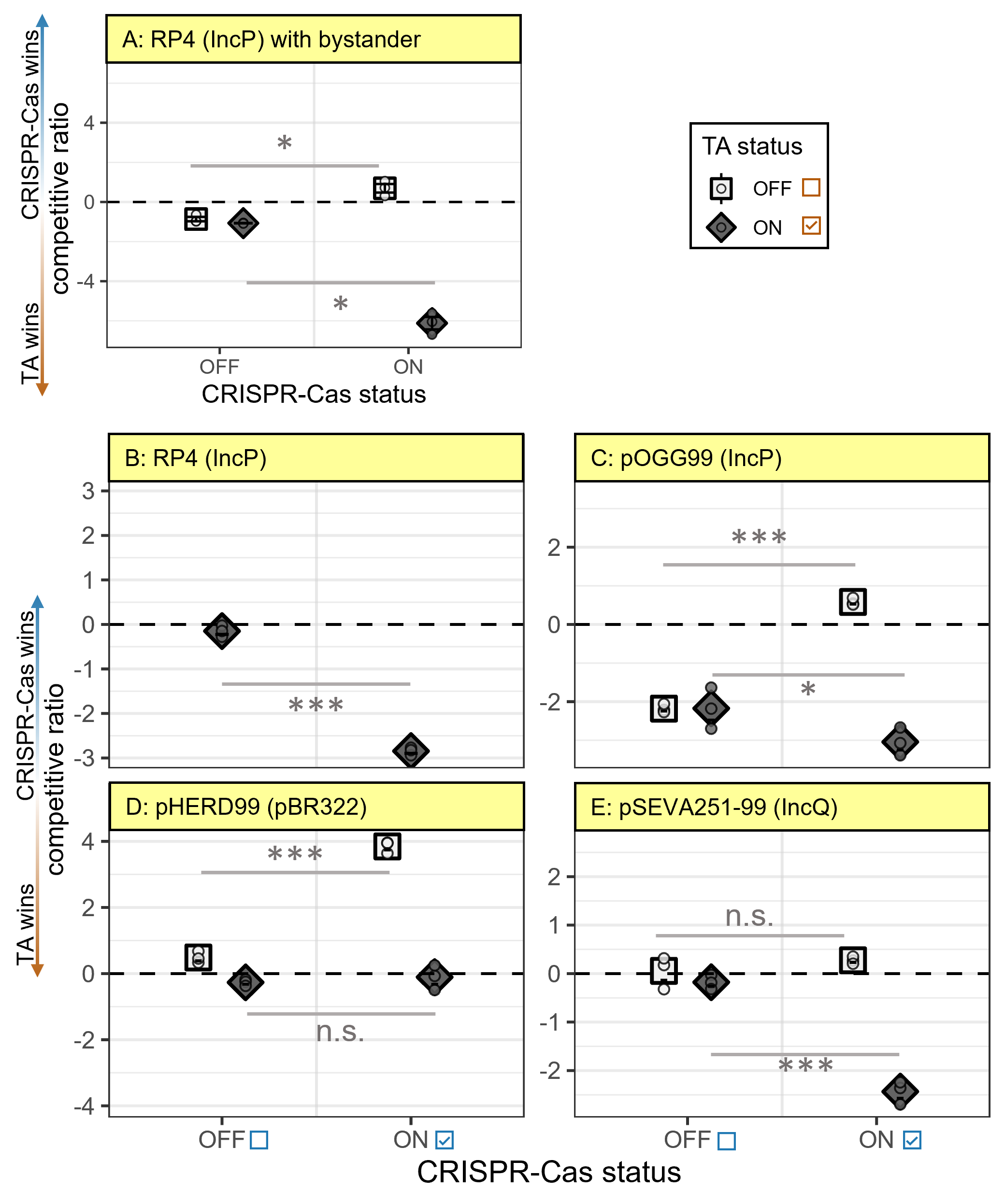

Supplement: S3 Fig — Mean ± standard error of the competitive ratio (log10 of pKJK5-carrying hosts/competitor-carrying hosts) describes the outcome of plasmid competition (N = 5). Values >0 indicate CRISPR-Cas plasmid pKJK5 winning the competition, and values <0 indicate the TA competitor plasmid winning the competition for a DH5α host where CRISPR-Cas was acting offensively. Data are presented for treatments in which CRISPR-Cas and TA activity were toggled on or off in all combinations. RP4 competition outcome (A, B) in this model system matched the outcome observed earlier (Fig 2B). Relative differences between treatments are indicated with gray lines and were assessed by Tukey’s HSD after fitting individual Generalized Linear Models; see Methods and Tables 6 and 7 for model details and p values. *p < 0.05; ***p < 0.001; n.s. not significant p > 0.63. The data underlying this Figure can be found in S1 Data. See S1 Text for additional information. (TIF) [file pbio.3003658.s003.tif]

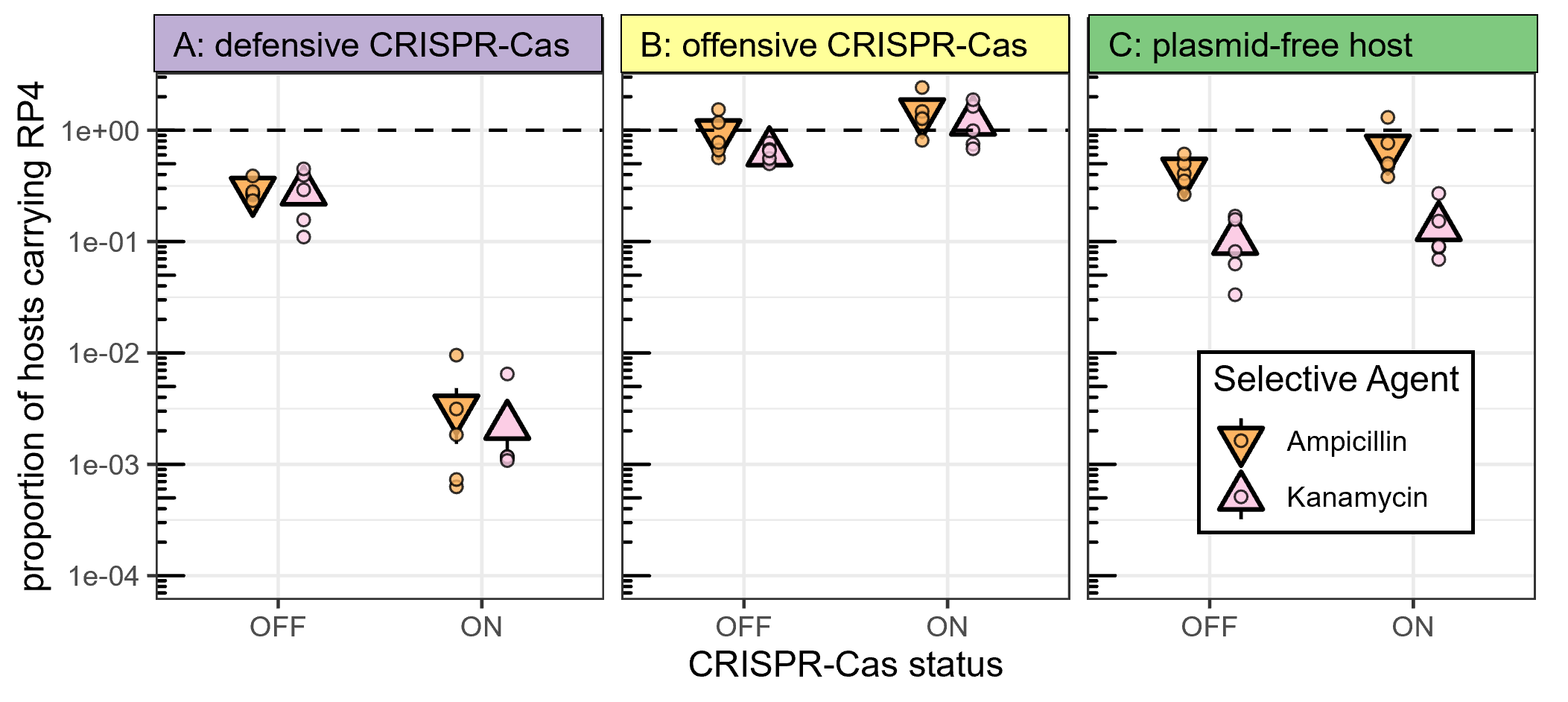

Supplement: S4 Fig — Mean and standard error of RP4 content of hosts in pilot mating experiment lacking a bystander plasmid assessed by ampicillin or by kanamycin, N = 5. Data are presented for CRISPR-Cas switched on or off. The data underlying this Figure can be found in S1 Data. See S1 Text for additional information. (TIF) [file pbio.3003658.s004.tif]

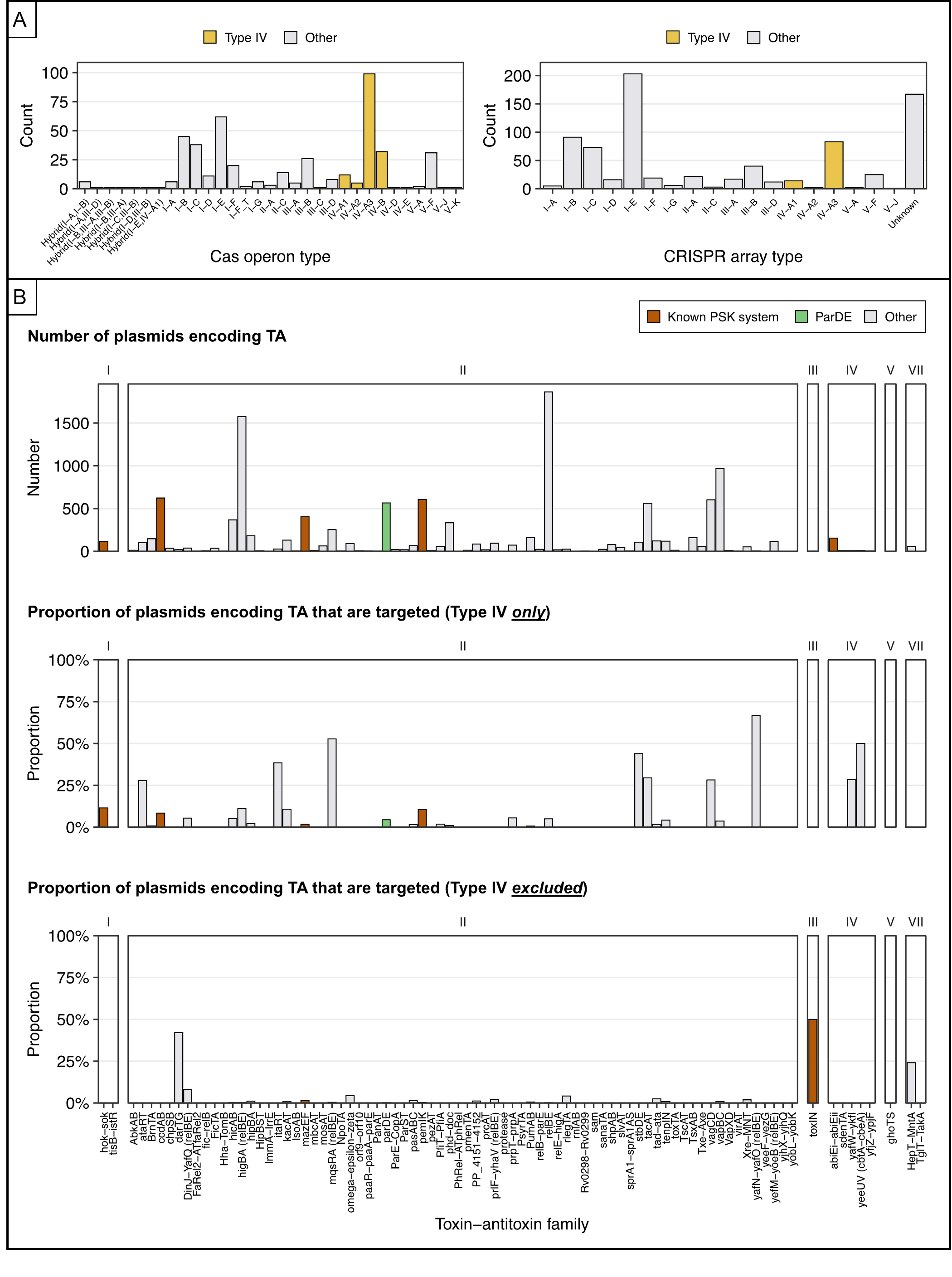

Supplement: S5 Fig — (A) CRISPR-Cas systems encoded on plasmids; split into distribution of Cas operon and CRISPR array types. Type IV systems are highlighted to emphasize their predominance. (B) The total number of plasmids encoding each TA family, highlighting parDE and other well-studied post-segregational killing (PSK) systems [13]. The other bar charts show the proportion of plasmids encoding each TA family targeted by plasmid borne Type IV and non-Type IV CRISPR-Cas systems. The data underlying this Figure can be found in S1 Data. (TIF) [file pbio.3003658.s005.tif]

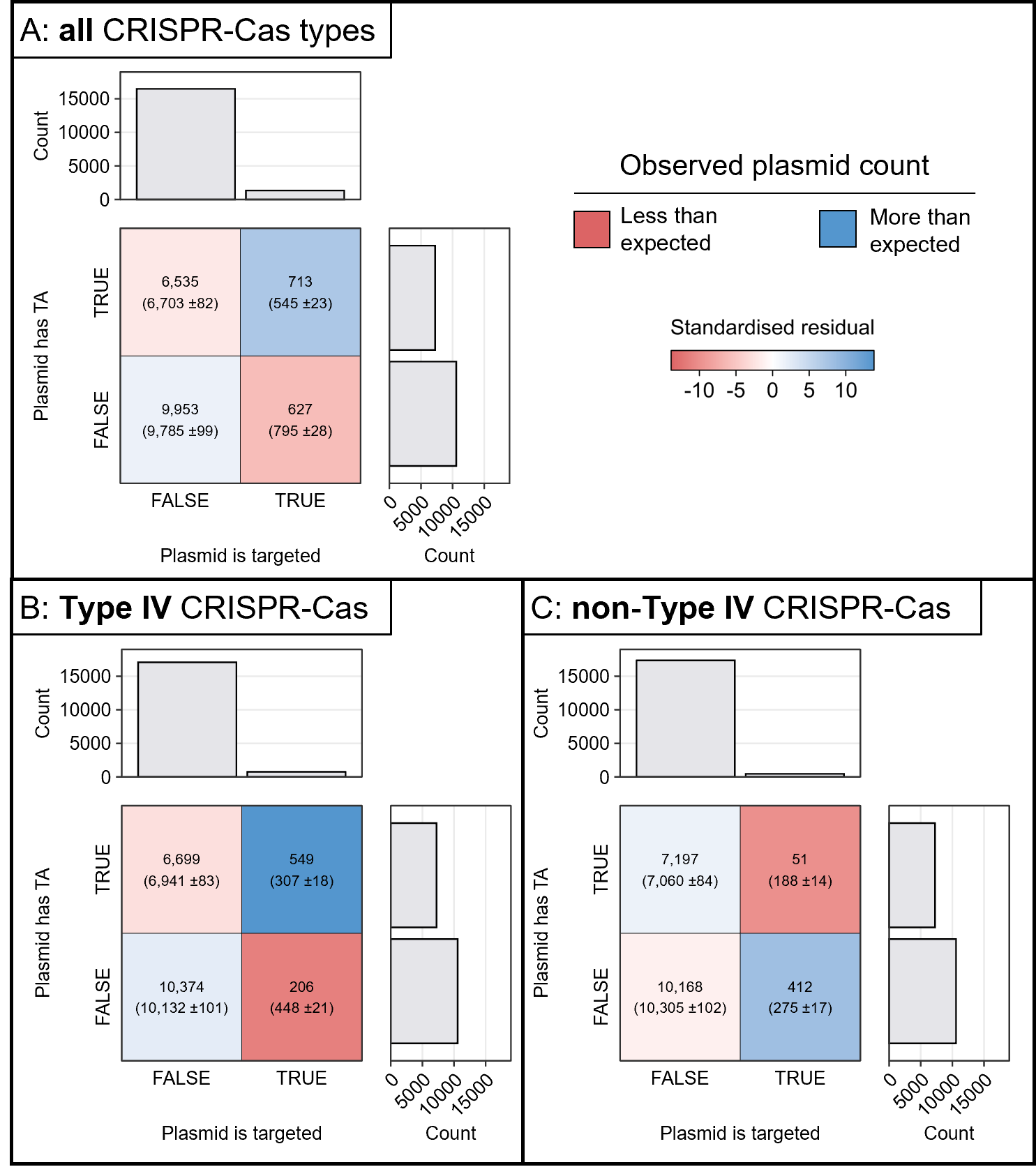

Supplement: S6 Fig — Contingency tables showing the relationship between plasmid targeting and TA presence. The tables display the observed counts of plasmids with or without TA systems and whether they are targeted or not by CRISPR spacers in another plasmid, either for all CRISPR-Cas types (A), Type IV CRISPR-Cas only (B), or for non-Type IV CRISPR-Cas (C). The bar plots show the sum of counts for each vertical or horizontal group. Facets in the table are coloured by standardized residual after Pearson’s χ2 test, indicating magnitude and directionality of association (red—less than expected, blue—more than expected). The data underlying this Figure can be found in S1 Data. (TIF) [file pbio.3003658.s006.tif]
