# Supplementary material for: CRISPR-Cas is beneficial in plasmid competition, but limited by competitor toxin–antitoxin activity when horizontally transferred
Source: PLoS Biol. 2026 Feb 19;24(2):e3003658. doi: 10.1371/journal.pbio.3003658 (PMC12945316; doi:10.1371/journal.pbio.3003658)
Supplement: S1 Text — Additional narrative and data analysis of S2–S4 Figs. Three-strain competition showed putative generalisability of CRISPR-Cas detriment in offense when facing TA (S2 Fig), the competitive outcome was not due to RP4’s backbone (S3 Fig), and measured RP4 proportions were robust to altering the selective agent to Ampicillin (S4 Fig). (DOCX) [file pbio.3003658.s007.docx]

**S1 Text: SUPPLEMENTARY RESULTS**

**CRISPR-Cas is beneficial in plasmid competition, but limited by competitor toxin-antitoxin activity when horizontally transferred.**

*PLOS Biology* 2026, https://dx.doi.org/10.1371/journal.pbio.3003658

David Sünderhauf*^1^, Jahn R. Ringger^1+^, Leighton J. Payne^2^, Rafael Pinilla-Redondo^2^, William H. Gaze^3^, Sam P Brown^4, 5^, Stineke van Houte*^1^

*corresponding authors; DS: [david@sunderhauf.net](mailto:david@sunderhauf.net), SvH: [C.van-Houte@exeter.ac.uk](mailto:C.van-Houte@exeter.ac.uk)

^1^ Environment and Sustainability Institute, University of Exeter Penryn Campus, Penryn, TR10 9FE, UK

^2^ Section of Microbiology, Department of Biology, University of Copenhagen, Copenhagen, Denmark

^3^ European Centre for Environment and Human Health, University of Exeter Penryn Campus, Penryn, TR10 9FE

^4^ School of Biological Sciences, Georgia Institute of Technology, Atlanta, Georgia, USA

^5^ Center for Microbial Dynamics and Infection, Georgia Institute of Technology, Atlanta, Georgia, USA

^+^current address: Department of Environmental Sciences, University of Basel, 4051 Basel, Switzerland

Three-strain competition shows putative generalisability of results

We repeated the two-strain competition experiment (Fig 2) using an additional plasmid host. In this way, we included an intermediate scenario where both plasmids invade an initially naïve, plasmid-free host, so that the mode of action of CRISPR-Cas could be either offensive or defensive. Here, we used DH5α::SmR as the initial CRISPR plasmid host, DH5α::CmR as the initial TA plasmid host, and DH5α::GmR as the naïve host (S2B Fig). A low limit of detection of TA plasmid RP4 in the defensive CRISPR-Cas host (S2C Fig) led to missing datapoints, especially where TA was turned off, and did not allow us to draw quantitative conclusions of the impact of TA status on the benefit conveyed by CRISPR-Cas in this host. Nonetheless, this analysis revealed that when CRISPR-Cas was acting defensively, the CRISPR-Cas plasmid pKJK5 won the competition in every case where CRISPR-Cas was switched on (Fig 2C; competitive ratio = 1.41 (N=1) and 1.94 ± 0.0085 respectively; significantly higher than 0 with p < 0.001 after T-test and Bonferroni adjustment for multiple testing with α = 0.005). In stark contrast, when CRISPR-Cas was acting offensively, switching on CRISPR-Cas had a differential effect depending on TA status of the competitor plasmid. When TA was absent from the competitor, switching on CRISPR-Cas brought little or no benefit to pKJK5 (S2D Fig; competitive ratio = 0.32 ± 0.13; p = 0.07). However, when the competitor plasmid’s TA was switched on, switching on CRISPR-Cas was clearly detrimental to pKJK5 (competitive ratio = -2.19 ± 0.11; p < 0.001).

In the naïve host, switching on CRISPR-Cas on its own benefitted pKJK5 (S2E Fig; competitive ratio 0.73 ± 0.1; p = 0.0019). When TA was switched on alongside this, there was no clear winner of the competition (competitive ratio = -0.21 ± 0.096; p = 0.1), however CRISPR-Cas still provided a benefit to pKJK5 relative to the scenario where CRISPR-Cas was switched off, where TA plasmid RP4 won the competition (competitive ratio = -0.94 ± 0.066; p < 0.001).

Overall, these data show that addition of a further plasmid host does not change the outcome of plasmid competition in hosts in which plasmids were already established.

Competitive outcome is not due to RP4’s backbone

TA plasmid RP4 is a 60kB conjugative plasmid carrying many gene cassettes, including additional TA systems. Therefore, we sought to independently verify any causal fitness effects of *parABCDE* in plasmid competition. We constructed three vectors with different backbones of varying incompatibility group (IncP, pBR322, and IncQ). We engineered each vector to carry RP4’s *parABCDE* operon and competed these with CRISPR-Cas plasmid pKJK5 delivered to recipients using an *E. coli* DH5α::SmR donor. As non-TA carrying controls, we used the respective empty vector backbones. These vectors were not self-transmissible, therefore we measured the outcome of plasmid competition when CRISPR-Cas was acting offensively only, described here as the competitive ratio within DH5α::CmR recipients. Firstly, the outcome of plasmid competition showed that previously observed results of competition with RP4 were largely upheld in this model system using a different DH5α tag variant as the CRISPR-Cas plasmid host, and also when lacking a bystander plasmid: switching CRISPR-Cas on in the presence of TA was detrimental to pKJK5 in offense (S3A-S3B Fig; p < 0.05 after fitting a GLM and a linear model, see Methods). When competing pKJK5 with vectors of varying incompatibility groups, we found that the TA system hindered the offensive ability of CRISPR-Cas on pKJK5. The competition outcome with pOGG99 (an IncP vector) directly mirrored our results recorded with RP4: turning on CRISPR-Cas in the absence of TA benefitted pKJK5, while turning on CRISPR-Cas in the presence of TA was detrimental to the CRISPR-Cas plasmid (S3C Fig; p < 0.001 and p = 0.47 in the absence and presence of TA respectively after fitting a GLM and carrying out Tukey’s post hoc test, see Methods). For the remaining two vectors (pBR322 backbone vector pHERD99 and IncQ backbone vector pSEVA251-99), the competitive ratios followed the same trend, and we observed a significant increase following CRISPR-Cas expression in favour of pKJK5 in the absence of TA for pHERD99 (S3D Fig, p < 0.001 after fitting a GLM and Tukey’s post hoc test), and a significant decrease, showing a detrimental CRISPR-Cas system, in the presence of TA for pSEVA251-99 (S3E Fig, p < 0.001 after fitting a GLM and Tukey’s post hoc test). The remaining comparisons between competitive ratios remained non-significant, but crucially CRISPR-Cas was never detrimental in the absence of TA, and never beneficial in the presence of TA.

The data generated with these simplified model systems strongly support our conclusion that RP4’s *parABCDE* operon limits CRISPR-Cas effectivity.

Measured RP4 proportion is robust to altering selective agent.

Throughout experimental work, RP4 carriage was assessed by selection using kanamycin, resistance to which is conferred by the CRISPR-Cas9 target gene *aphA*. This means theoretically, RP4 escape mutants with non-functional *aphA* genes could skew RP4 carriage results. However, plasmid escape of CRISPR-Cas by target mutation is unusual [1]. We carried out a control experiment in which we mated strains exactly as in S2A-S2B Fig, with the exception of omission of bystander plasmid pCDF1b (i.e. TA was active in all treatments). We confirmed that the outcome of RP4 carriage was largely robust to altering the selective agent to ampicillin (resistance is conferred by *blaTEM* on the opposite side of *aphA* on RP4, >25kB away). RP4 carriage was assayed as moderately lower when plating on kanamycin (10.11±2.68 – 13.44±3.69%) than when plating on ampicillin (42.72±6.0% – 68.64±16.75% for non-targeting and targeting treatments respectively) in only the DH5α::GmR host (S4C Fig), but this was observed for both pKJK5::csg treatments (S4 Fig). Therefore, these discrepancies are likely due to discrepancies in resistant colonies when using different combinations of antibiotics, and we infer that RP4 escaping pKJK5::csg[aphA99] targeting by *aphA* mutation is negligible in this work.

**References**

1. Jiang W, Maniv I, Arain F, Wang Y, Levin BR, Marraffini LA. Dealing with the Evolutionary Downside of CRISPR Immunity: Bacteria and Beneficial Plasmids. Matic I, editor. PLOS Genet. 2013;9: e1003844. doi:10.1371/journal.pgen.1003844
