# Supplementary material for: CRISPR-Cas is beneficial in plasmid competition, but limited by competitor toxin–antitoxin activity when horizontally transferred
Source: PLoS Biol. 2026 Feb 19;24(2):e3003658. doi: 10.1371/journal.pbio.3003658 (PMC12945316; doi:10.1371/journal.pbio.3003658)
Supplement: S2 Text — Development of the mathematical model (Fig 1) including derivation of equations used, and mathematical analysis of the impact of changing competitive traits x and y on plasmid transition rates and the outcome of plasmid competition. (DOCX) [file pbio.3003658.s008.docx]

**S2 Text: SUPPLEMENTARY MATHEMATICAL ANALYSIS**

**CRISPR-Cas is beneficial in plasmid competition, but limited by competitor toxin-antitoxin activity when horizontally transferred.**

*PLOS Biology* 2026, https://dx.doi.org/10.1371/journal.pbio.3003658

David Sünderhauf*^1^, Jahn R. Ringger^1+^, Leighton J. Payne^2^, Rafael Pinilla-Redondo^2^, William H. Gaze^3^, Sam P Brown^4, 5^, Stineke van Houte*^1^

*corresponding authors; DS: [david@sunderhauf.net](mailto:david@sunderhauf.net), SvH: [C.van-Houte@exeter.ac.uk](mailto:C.van-Houte@exeter.ac.uk)

^1^ Environment and Sustainability Institute, University of Exeter Penryn Campus, Penryn, TR10 9FE, UK

^2^ Section of Microbiology, Department of Biology, University of Copenhagen, Copenhagen, Denmark

^3^ European Centre for Environment and Human Health, University of Exeter Penryn Campus, Penryn, TR10 9FE

^4^ School of Biological Sciences, Georgia Institute of Technology, Atlanta, Georgia, USA

^5^ Center for Microbial Dynamics and Infection, Georgia Institute of Technology, Atlanta, Georgia, USA

^+^current address: Department of Environmental Sciences, University of Basel, 4051 Basel, Switzerland

Model development

Fig 1AB outlines the basic conceptual assumptions underpinning our model. Our mathematical model focusses on the two classes of co-infected cells (*CT* and *TC*), and defines transition rates from these co-infected states to singly infected states (*C* or *T*).

These transition rates are defined by plasmid segregational loss (baseline rate *s*), modified by *C-* and *T-*specific traits of CRISPR-Cas induced enhanced segregation (multiplicative factor *x*; *x* > 1 implies increased segregation rate *xs > s*) and TA-induced PSK (probability of PSK, *y*). In the event of PSK, we assume that the liberated resources (nutrients, space) benefit a kin cell weighted by parameter *f_r_*, benefit a non-kin cell (*f_i_*), or benefit no cells (e.g. due to cell dormancy versus PSK; *f_0_*), where *f_0_* + *f_i_* + *f_r_* = 1 [1]. Finally, asymmetries in parameter values between resident and invader roles are captured by subscripts *r* and *i*. Given lower plasmid copy number and lower gene expression for invading versus established plasmids [2], our default assumptions are *s_i_* > *s_r_*; *x_r_* > *x_i_* ≥ 1; 1 > *y_r_* > *y_i_* ≥ 0. Given spatial structuring so that *T* cells are enriched in neighbourhood of *TC* cells (and vice-versa for *C* near *CT* cells), we further assume 1 > *f_r_* > *f_i_* ≥ 0.

Transitions for *CT* cells (when the CRISPR-Cas plasmid is resident) are formalised as follows.

$t_{CT,C}={x_{r}s}_{i}{((1-y}_{i})+y_{i} f_{r})$ E1; for transitions to CRISPR-Cas plasmid *C.*

$t_{CT,T}=s_{r}+x_{r} s_{i} y_{i} f_{i}$ E2; for transitions to TA plasmid *T*.

Similarly, transitions for *TC* cells (when the TA plasmid is resident) are formalised below.

$t_{TC,C}=x_{i}s_{r}(\left( 1-y_{r} \right)+y_{r} f_{i})$ E3; for transitions to CRISPR-Cas plasmid *C.*

$t_{TC,T}=s_{i}+x_{i} s_{r} y_{r} f_{r}$ E4; for transitions to TA plasmid *T.*

Mathematical analysis.

Analysis of how the transition rates (equations E1-E4) change with competitive traits *x* and *y* reveals the following predictions:

1. In the absence of PSK (*y* = 0), increasing investment in CRISPR-Cas (increasing *x*) always increases transition gains, regardless of whether the CRISPR-Cas plasmid is in a resident or invader role:

${\frac{d t_{CT,C}}{d x_{r}}|}_{y_{i}=0}=s_{i} > 0$ ; ${\frac{d t_{TC,C}}{d x_{i}}|}_{y_{r}=0}=s_{r}> 0$

1. In the absence of CRISPR-Cas (*x* = 1), increasing investment in TA-mediated PSK (increasing *y*) always increases *T*-plasmid gains, again regardless of role:

${\frac{d t_{CT,T}}{d y_{i}}|}_{x_{r}=1}={f_{i}s}_{i} > 0$ ; ${\frac{d t_{TC,T}}{d y_{r}}|}_{x_{i}=1}={f_{r}s}_{r}> 0$

1. When both are active (*x* > 1 and *y* > 0), an asymmetry emerges between TA and CRISPR-Cas. TA-mediated transition gains (*t_TC,T_, t_CT,T_*) are increasing functions of CRISPR-Cas activity *x* ($\frac{d t_{CT,T}}{d x_{r}}={f_{i}s}_{i} y_{i}>0;\frac{d t_{TC,T}}{d x_{i}}={f_{r}s}_{r}y_{r}>0)$, while CRISPR-Cas-mediated gains are *decreasing* functions of TA activity *y* ($\frac{d t_{CT,C}}{d y_{i}}={{- x}_{r}s}_{i}\left( 1-f_{r} \right)<0;\frac{d t_{TC,C}}{d y_{r}}={{- x}_{i}s}_{r}\left( 1-f_{i} \right)<0)$: PSK destroys the advantages of competitor segregation, regardless of resident or invader role.

To investigate how increasing investment in CRISPR-Cas (*x*) impacts competitive outcomes (CRISPR gains versus TA gains), we analyse outcomes using differences in mutually exclusive transition paths Δ*_C_*, from a defined ecological context (*CT* or *TC*, Fig 1AB, equations E1-E4). To begin, we examine the ‘resident CRISPR-Cas’ context (*CT* cells), and define the resident CRISPR-Cas Δ*_Cr_* as

$\Delta_{Cr}= t_{CT,C}- t_{CT,T}= -s_{r}+ s_{i}x_{r}(1-y_{i}\left( 1+f_{i}-f_{r} \right))$ E5

When Δ*_Cr_* > 0, *CT* cells resolve to *C* cells more often, compared to *T* cells. When Δ*_Cr_* < 0, resolution to *T* cells dominates. We next examine how Δ*_Cr_* changes with increasing CRISPR-Cas investment, *x_r_*:

$\frac{d \Delta_{Cr}}{d x_{r}}=s_{i} (1-y_{i}\left( 1+f_{i}-f_{r} \right))$ E6

From equation E6 we can see that in the absence of invader TA activity (*y_i_* = 0), $\frac{d \Delta_{Cr}}{d x_{r}}=s_{i}$ reinforcing that resident CRISPR-Cas investments *x_r_* are always beneficial in absence of TA activity. When TA is active, we see that the return on investment ($\frac{d \Delta_{Cr}}{d x_{r}}$) is decreasing with *y_i_*. While decreasing, we note that given our assumptions *f_r_* ≥ *f_i_* and 0 ≤ *y_i_* ≤ 1, equation E6 cannot turn negative. For our default parameters (see parameterization table below), maximum *y_i_* = 0.66, *f_i_* = 0.04 and *f_r_* = 0.2, and therefore $y_{i}\left( 1+f_{i}+f_{r} \right)=0.66\times0.84=0.55$.

We next examine the context when the CRISPR-Cas plasmid is an invader (*TC* cells), and define the competitive outcome in this context Δ*_Ci_* as

$\Delta_{Ci}= t_{TC,C}- t_{TC,T}= -s_{i}+ s_{r}x_{i}(1-y_{r}\left( 1-f_{i}+f_{r} \right))$ E7

When Δ*_Ci_* > 0, *TC* cells resolve to *C* cells more often, compared to *T* cells. When Δ*_Ci_* < 0, resolution to *T* cells dominates. We next examine how Δ*_Ci_* changes with increasing CRISPR-Cas investment, *x_i_*:

$\frac{d \Delta_{Ci}}{d x_{i}}=s_{r} (1-y_{r}\left( 1-f_{i}+f_{r} \right))$ E8

From equation E8 we can again see that in the absence of TA activity (*y_r_* = 0), CRISPR-Cas is always beneficial $\frac{d \Delta_{Ci}}{d x_{i}}=s_{r}$. When TA is active, we see that the return on investment ($\frac{d \Delta_{Ci}}{d x_{i}}$) is decreasing with *y_r_*, and can turn negative if $y_{r}\left( 1-f_{i}+f_{r} \right)>1$. This condition is possible given our assumption that *f_r_* > *f_i_*, and is favoured by our assumption *y_r_* > *y_i_*. Indeed, we find this condition to be met for our default parameters. For these defaults, *y_r_* = 0.99, *f_r_* = 0.2 and *f_r_* = 0.2, and therefore $y_{r}\left( 1-f_{i}+f_{r} \right)=0.99\times1.16=1.15$.

References

1. Rankin DJ, Turner LA, Heinemann JA, Brown SP. The coevolution of toxin and antitoxin genes drives the dynamics of bacterial addiction complexes and intragenomic conflict. Proc R Soc B Biol Sci. 2012;279: 3706–3715. doi:10.1098/rspb.2012.0942

2. Fraikin N, Couturier A, Lesterlin C. The winding journey of conjugative plasmids toward a novel host cell. Curr Opin Microbiol. 2024;78: 102449. doi:10.1016/j.mib.2024.102449
