# Supplementary material for: CRISPR-Cas is beneficial in plasmid competition, but limited by competitor toxin–antitoxin activity when horizontally transferred
Source: PLoS Biol. 2026 Feb 19;24(2):e3003658. doi: 10.1371/journal.pbio.3003658 (PMC12945316; doi:10.1371/journal.pbio.3003658)
Supplement: Attachment — Submitted filename: plascomp_reviewer_comments.docx [file pbio.3003658.s010.docx]

**Please note that all line numbers have been updated to refer to our latest manuscript version with tracked changes and “show all markup”.**

**Reviewer #1:**

The authors investigate the competition between a plasmid carrying a CRISPR-Cas defense system and one carrying a toxin-antitoxin (TA) system. This is a biologically interesting scenario, as CRISPR-Cas systems can target and degrade competing plasmids, while TA systems typically induce cell death upon plasmid loss, due to the greater stability of toxins relative to antitoxins. To explore this, the authors construct a toy model of plasmid competition, validate predictions with experiments, and perform a bioinformatic analysis of plasmid co-occurrence.

The manuscript is generally well-written, the figures are clear, and the experimental data are presented effectively. Although the dataset is limited in size, I do not view this as problematic provided the conclusions are robust and well-supported. However, I find several key interpretations unconvincing, both in terms of the modeling and the biological conclusions. If my concerns are valid, they undermine the central claims of the manuscript. I would welcome clarification, but in its current form I do not consider the work suitable for publication.

We thank the reviewer for their comments. We have taken on board their concerns regarding the match of modelling predictions, experimental data, and bioinformatic analyses and address these below by changing the way we present our model output to emphasise our qualitative results.

1. Discrepancies between model predictions and experimental results:

The authors assert strong agreement between theoretical predictions and experimental results. However, several inconsistencies suggest otherwise:

We have previously stated the following core predictions of our mathematical model (L135 – 144):

1) In the absence of TA, CRISPR-Cas increases competitive success
2) In the presence of TA, CRISPR-Cas is beneficial when resident, but detrimental when offensive.

These core predictions, which we visualised in Figure 1BC of the original version of the manuscript, and mathematically derived in SI, are also reflected in our experimental results. Thanks to these comments, we now realize we had not successfully differentiated between a set of core predictions that our experiment was designed to test, and other more peripheral predictions that can be extracted from our model, but our existing data is not positioned to test. To address this misunderstanding, we have improved our signposting. In the experimental results L249, we now specify that our experimental data is “in agreement with our **core** modelling predictions”, and in the discussion L369 we now state “Our experimental model system showed the same outcome **as these core modelling predictions**”. Predictions beyond these are not the focus of our model, and we further clarify this below.

1. In Fig. 2C, the CRISPR-Cas system has a strong effect when TA is present and a weak or no effect when TA is absent. However, the model (Fig. 1B) predicts the opposite: the effect of CRISPR-Cas (measured as the difference between CRISPR-Cas strength = 0 vs >0) is greatest when TA is absent. This contradicts the experimental results.

The reviewer is right that this is a prediction from our model. We now clarify that (1) this prediction does not form a core prediction of our model, and (2) our experiment is not designed or able to test this prediction. Addressing point (2) has led to changes in our data presentation in Fig 2C to make this point clear. Experimentally, in the relevant scenario (Figure 2C), we cannot make any quantitative comparison between the competitive ratio in the absence and in the presence of TA. In the absence of TA, we only detect TA plasmids in 1 out of 5 replicates (both for CRISPR-Cas-off and -on treatments). The corresponding means presented in Figure 2C are therefore based on a single replicate and marked *na* (statistically not assessed). These means represent minimum theoretical competitive ratio values. Therefore, while we are confident that CRISPR-Cas provides a benefit to the plasmid that bears it, we intentionally do not make any quantitative statements on whether this benefit is greater in the presence or absence of TA.

In order to make clear that these do not form core predictions of our model, and that these specific results suffer from a low limit of detection resulting in a reduced *n*, we have adjusted the opacity of the datapoints in Figure 2C, added a sentence explaining this in the results L219-222, and adjusted the summary of this section by clarifying “in agreement with our **core** modelling predictions” in L249.

2. The authors claim that CRISPR-Cas is highly detrimental in the presence of a resident TA plasmid, and this is observed experimentally (Fig. 2D). However, the model (Fig. 1C, black line) shows only a small decrease in survival, suggesting minimal detrimental effect.

We have adjusted the claim of highly detrimental to “clearly detrimental” (L231), as this treatment combination shows a clear detriment in comparison to the matching TA-off scenarios which no significant benefit or detriment to switching on CRISPR-Cas. In addition, we have completely reworked Figure 1 (along with substantial changes to the SI), with the goal to emphasise the *qualitative* predictions of our model (is CRISPR-Cas beneficial or detrimental in a given context?), and to reduce any implied quantitative predictions on magnitudes of effect. These changes have reduced the number of model parameters and associated assumptions, and increased our focus on qualitative predictions, leading to qualitative tests.

3. On closer inspection, the model implies that CRISPR-Cas is always beneficial for the resident plasmid, especially when the invading plasmid lacks or has a weak TA system. Overall, the dominant factor appears to be whether TA is present or absent, rather than the presence of CRISPR-Cas.

These are our conclusions, too. When resident, CRISPR-Cas is always beneficial. In offense, CRISPR-Cas is detrimental in the presence of TA.

2. Interpretation and implementation of the bioinformatic analysis:

The authors hypothesize that co-existence between TA and CRISPR-Cas systems should be rare due to the model's predicted competition, and they investigate this via a plasmid database.

As discussed above, I do not believe the model robustly predicts a strong anti-correlation, undermining the rationale for this hypothesis.

Our substantial revisions of the modelling sections clarify the conditions where investments in CRISPR-Cas systems are disfavoured, given competition with resident TA plasmids with active PSK systems (high *y_r_*) and sufficient spatial structure (*f_r_* > *f_i_*), see equation E7. These results provide a logical basis for our bioinformatic prediction that TA plasmids will limit the prevalence of CRISPR-Cas plasmids targeting TA plasmids.

4. The exclusion of Type IV CRISPR systems from the analysis is insufficiently justified. Although Type IV systems are thought to suppress gene expression rather than cleave DNA, suppression of antitoxin expression would likely still trigger toxin-mediated cell death. Reference [8], which the authors cite to justify this exclusion, does not appear to address TA systems and does not support this decision.

Reference [8] supports the statement that Type IV systems suppress gene expression and cause plasmid persistence despite targeting. This phenomenon is well-established, and we have added an additional reference [29] to evidence this (L299). Indeed, targeting of antitoxins would still trigger toxin-mediated cell death, but targeting of any other plasmid gene (e.g., antibiotic resistance genes, as in the experiment, or plasmid conjugation genes, as frequently observed in nature) would not. We carried out an additional analysis of locations of Type IV CRISPR-Cas targets within our dataset, and found no instances of antitoxin genes being targeted, and no signal of preferential targeting in proximity to TA systems. This shows that, within our dataset, putative suppression of antitoxins by Type IV CRISPR-Cas is not relevant. Please also see our response to Reviewer 2, point 3.

5. Most critically, the model and experiments do not permit co-existence of TA and CRISPR-Cas plasmids — they are framed in terms of competition between mutually exclusive plasmids. Yet the bioinformatic analysis examines co-occurrence in the same cell. As such, the model and experiments do not directly inform expectations for plasmid co-existence in natural populations.

On the contrary, our model does permit coexistence states. Indeed, our initial states are cells experiencing co-infection (*CT* and *TC* cells). Our model does assume that these coexistence states are not permanent and tracks their resolution into *C* or *T* cells. To address this comment, we have reworked schematic Figures 1AB to better illustrate the competing transition paths from the initial *CT* and *TC* states.

Our bioinformatic analysis examines which plasmid-borne CRISPR-Cas spacers target other plasmids – i.e., we build a picture of past plasmid co-infections. We cannot make any statement about current coexistence. Our model, experiment, and bioinformatic analysis all investigate the situation in which one plasmid with a CRISPR-Cas system targets another plasmid while they are present within the same cell.

We expand on the experimental system in Figure S2 to investigate compatible plasmids that permit co-existence and find that our key results are upheld. These experiments have now been repeated with the same *Escherichia coli* strains as used in the main body of the manuscript; please also see our response to Reviewer 2, point 1.

6. Furthermore, CRISPR-Cas systems would not be expected to target co-resident TA plasmids, which would presumably be recognized as self. Thus, the connection between the first two figures and the database analysis is tenuous.

Plasmid-borne CRISPR-Cas systems would be expected to acquire a spacer from a co-resident TA plasmid, followed by targeting the plasmid. CRISPR-Cas systems do not have further self/non-self recognition mechanisms. Perhaps we misunderstand this question?

Conclusion:

In summary, while the study addresses a compelling biological question and employs an integrative approach, I find major issues in the interpretation of both the model and the bioinformatic analysis. These concerns raise doubts about the central conclusions of the work. Unless the authors can convincingly resolve these points, I do not consider the manuscript suitable for publication.

We appreciate the reviewer’s concerns, and in light of both reviewers’ invaluable comments reworked the presentation of our mathematical model to emphasise its qualitative predictions. In addition, we have now highlighted limitations of our experimental data, and included additional bioinformatic and experimental analyses which provide further evidence for the differential action of Type IV and non-Type IV CRISPR-Cas systems as well as competition of plasmids with compatible backbones.

**Reviewer #2:**

The authors explore the benefits of carrying CRISPR-Cas and TA systems in plasmid competition using mathematical modelling, experiments and bioinformatic analysis. The study shows that the benefit of competition systems is dependent on the ecological context, invader versus resident. CRISPR-Cas was shown to be a generally beneficial strategy for resident plasmids but become detrimental when trying to invade a population of TA-system carrying plasmids. The bioinformatic study suggests that these effects are dependent on the CRISPR-Cas type.

The study of plasmid competition and interactions is timely and exciting. Overall, the combination of experiments, modelling and bioinformatic analysis comprehensively supports the conclusions. The manuscript is well written, and I would like to particularly applaud the diligent methods description.

We thank the reviewer for their kind words and helpful comments. In summary, to address these comments, we have 1) reworked our theoretical model, 2) conducted additional control experiments, and 3) included further bioinformatic analyses, which we expand on below.

Major comments:

1. IncP1 plasmids contain entry exclusion systems and belong to the same plasmid incompatibility group. This will drastically lower the rate of coinfection as compared to non-related plasmids and potentially lead to heterogeneity in plasmid carriage within the population. How does the addition of these competitive strategies affect the conclusions about CRISPR versus TA systems? It seems that this is being addressed to some extent in Fig. S4, but this needs more explanation and the comparison with Fig. 2 is made a bit difficult because E. coli K12 was used as a donor instead of E. coli DH5a. Conjugation rates with these two donors could vary substantially, given that DH5a has a reduced genome making it more suitable for molecular cloning and transformation.

We agree that entry exclusion is a mechanism which can reduce the rate at which the plasmids encounter each other, but we hypothesise that this means our results speak even more strongly, as we observe differential plasmid competition dynamics even if co-infection rates may be low.

We took on board the reviewer’s comments regarding the use of different *E. coli* strains and repeated the experiments using the same *E. coli* DH5a strains from the main manuscript to strengthen this link, please see our updated Figure S2. In brief, in all cases, turning on CRISPR-Cas in the absence of TA is beneficial (or has no effect), and turning on CRISPR-Cas in the presence of TA is detrimental (or has no effect), which confirms our results. It is worth noting that, nonetheless, this simplified model system varies from the three-strain competition presented in the main manuscript, as the naïve *E. coli* strain is absent. This is reflected in the quantitative differences to the main manuscript observed for competition with RP4, even in presence of a bystander plasmid (Figure S2A).

We further rephrased the results L239-241 to read “In addition, to determine whether our observed effects are driven by RP4’s *parABCDE* TA system rather than other RP4-encoded mechanisms **such as entry exclusion or incompatibility…**”. This clarifies the rationale behind carrying out the additional experiment.

2. While I agree with the general idea behind the parameter f and the potential benefit of PSK for surrounding cells if cell lysis occurs, I am not entirely sure that it is justified for this study. The experimental system is using a TA system that works through DNA gyrase inhibition, which results in a more bacteriostatic effect and, if lysis occurs at all, probably with a substantial time delay. Rather, these TA systems can lead to SOS response activation and cell filamentation, which would result in a neutral to negative effect on surrounding cells.

Thank you very much for this comment, which is an important point in aligning our experiments with the model that we hadn’t considered. It is true that our model relies on a positive effect to nearby cells after TA activity via *f*, but we believe that this is nonetheless the case with RP4’s *par* TA system, which we expand on below. It is additionally worth noting that our chosen model parameters *f_i_* + *f_r_* < 1 assume that only a minority of instances of TA activity lead to a benefit to nearby cells, while the majority of instances (*f_0_* = 1 - *f_i_* - *f_r_* = 0.76) give no benefit to any cell type, which implies a partially bacteriostatic or non-beneficial effect of TA activity. Together with reworking the presentation of our modelling output, we have now explicitly included this metric *f_0_* in Figure 1AB, and explain this in SI L35-37.

In addition to the bacteriostatic effect of cell filamentation, DNA gyrase inhibition by ParE leads to cell death. We have now clarified this in the manuscript L198-199. Specifically, Roberts *et al.* 1994 *JMB* show that removal of *parDE* from cells leads to a marked decrease in viable cells (assessed by plating) compared to total cells (assessed by microscopy) within 6 hours, in addition to an increase in the proportion of filamentous cells. Please compare particularly Roberts *et al.* 1994 *JMB* Figure 6 (reproduced in reviewer supplement Figure R1).

Furthermore, we hypothesise that cell filamentation can also impose a positive effect on nearby kin cells, rather than a neutral to negative effect. Fraikin *et al.* carried out visualisations of *E. coli* cells experiencing ParE activity (Fraikin & van Melderen 2024 *NAR*). Firstly, they anecdotally observed instances where filamenting cells experience loss of cytosolic content followed by cell death, although this is not quantified. More importantly, cells filamenting due to ParE activity take up substantially less space than control cells after 16 hours of growth; compare especially Fraikin & van Melderen 2024 *NAR* Supplementary Movie 1 (control cells) and Supplementary Movie 5 (*parDE* cells), representative screenshots reproduced in reviewer supplement Figure R2. This additional space is a resource nearby kin cells not experiencing filamentation could exploit.

Finally, we sought to directly show increased levels of cell death as a result of CRISPR-Cas and TA activity in our own experiments. To minimise confounding variables, we carried out Live-Dead staining using samples derived from the two-strain experiment (Figure S2; redone based on Reviewer 2 Comment 1). We opted for this experiment rather than the three-strain experiment presented in the main manuscript due to absence of the naïve strain (where not all instances of conjugation will lead to co-occupation by RP4 and pKJK5), and due to the samples from the three-strain experiment being in cryogenic storage since September 2023, which would lead to excessive background cell death.

In brief, we stained samples obtained from the experiment presented in Figure S2A using Propidium Iodide, which specifically stains dead cells red. First, some single-strain controls showed a higher level of background cell death than expected (reviewer supplement Figure R3A). While we do not currently have an explanation for this phenomenon, we believe that disentangling the mechanistic basis for background cell death in the single-strain controls (particularly when culturing cells containing RP4) is beyond the scope of this paper.

Crucially however, in the two-strain experiment, we observed that when TA was turned on, switching on CRISPR-Cas significantly increased the fraction of dead cells. Such an increase was not observed when TA was turned off (reviewer supplement Figure R3B), thus showing a clear CRISPR-Cas- and TA-dependent increase in cell death in our experimental model system.

In summary, we addressed this concern in our manuscript by now explicitly clarifying that a result of no benefit to surrounding cells of TA activity was implied in our model, as well as providing additional literature evidence.

3. There are a few interesting points in the bioinformatic analysis that might be worth exploring further and would strengthen the argument:
   - First, it wasn't mentioned if CRISPR-Cas carrying plasmids do also carry TA systems? Or potentially they even just carry anti-toxins?

Indeed, particularly non-Type-IV CRISPR-Cas-bearing plasmids could benefit from TA or antitoxin carriage and agree that this is worth investigating. We carried out an additional analysis and found that nucleolytic non-Type-IV CRISPR-Cas-bearing plasmids carry TA systems that match the family of TA systems found on plasmids they target. This strengthens our findings, and is now presented in Figure 3C and L318-331, and in the discussion L374-375.

Interestingly, we did not find orphan antitoxin genes to be relevant. They are included in our analysis, but none of the non-Type IV CRISPR-Cas plasmids carry an orphan antitoxin of matching family, and only 37 out of 352 Type IV CRISPR plasmids carry matching orphan antitoxins.

- Second, it looks like Type IV CRISPR targeting of TA carrying plasmid is higher than expected. It would be interesting to see if Type IV CRISPR target specifically the toxin gene of the TA system, so that segregational loss of the plasmid is not detrimental anymore.

We were also surprised to see a positive association between Type IV CRISPR-Cas targeting and TA carriage. Type IV CRISPR-Cas systems are hypothesised to affect gene expression within ~2-5kB of their target sequence (Benz *et al.* 2024 *Cell Host Microbe*; Sanchez-Londono *et al.* 2024 *NAR*). We investigated whether toxin genes are specifically being targeted in our dataset, and found no association. Within 5kB of a TA system on a target plasmid, there is no pattern of preferential targeting by Type IV plasmid-borne CRISPR-Cas systems, and indeed only 16% of Type IV targets fall into this 5kB proximity window on target plasmids (see reviewer supplement Figure R4).

More broadly, we hypothesise that the positive association of Type IV CRISPR-Cas targeting and TA carriage is due to confounding factors: plasmids that are more likely to compete with each other are expected to have similar life-history traits such as size and genetic cargo. TA can be beneficial in plasmid competition, therefore plasmids in active competition (which are being targeted by Type IV CRISPR-Cas) are likely to carry TA systems.

- Third, the authors investigated CRISPR-Cas types, but looking also into TA system types and their potential to lyse cells (rapidly) due to PSK could indicate the relevance of actual lysis or just cell death.

We considered focussing on specific TA systems, or partitioning TA systems according to Type or mode of action for our analysis, but unfortunately our dataset is not large enough to allow this analysis. Instead, in Figure S4 we highlight TA systems known from literature to elicit PSK, which constitute a minority of the dataset.

4. Selective plating for RP4 using kanamycin, which is also the target of the CRISPR spacer, might be misleading if the plasmids lose kanamycin to escape CRISPR but still manage to persist.

We had the same concern and had assessed this in a control experiment with a similar experimental setup. We have now added the data into our supplement (Figure S3) and indicated this in the Methods section (L577-579). In brief, we repeated selection for RP4 using ampicillin instead of kanamycin and found no escape from CRISPR-Cas9 by loss of *aphA*.

Minor comments:

5. L**33**: Given the title and the following sentence it is a bit unclear here if that is a general statement and how it relates to TA systems. The TA systems then come out of nowhere in the next sentence.

We understand the reviewer’s point, we have rephrased and included an introductory sentence on TAs in L25-26

6. L**30**: maybe 'defends its host' might be clearer than 'remains' ('remains' might trigger the question, as opposed to doing what?)

As plasmids acting defensively and offensively is not a common concept, we are trying not to introduce this in the abstract to keep it more generally understandable to a broad group of readers. We have rephrased to “when the plasmid has not recently transferred to a new host”.

7. L**51-53**: this makes it sound like the difference between chromosomal versus MGE encoded immune systems will be investigated

We have now rephrased this sentence to place a focus on the unique selection pressures faced by MGE-encoded immune systems.

8. L**87**: host population

This sentence makes a general statement of the selection pressure a CRISPR-Cas encoding plasmid faces. As modelled in the first section of our manuscript, this selection pressure is faced by an individual plasmid invading an individual new host cell. We therefore believe that leaving this sentence as “when CRISPR-Cas-bearing plasmids are acting offensively and invading a new host” is more accurate than specifying “invading a new host population”.

9. L**422-429**: it is a bit unclear here how co-opting the host's spacer machinery will help in overcoming TA activity

If a CRISPR-Cas-bearing plasmid does not already target a competitor plasmid, it cannot acquire a spacer targeting this competitor as readily when it is acting offensively compared to defensively. Hence, TA activity would be avoided by not targeting the TA plasmid. We have added an explainer “when moving to a new host, the CRISPR-Cas system cannot acquire new spacers until it is expressed and therefore cannot target a newly encountered plasmid already present in the new host” L426-429.

10. Figure 1 and SI: What is the benefit of adding the baseline fitness parameter a and transition rate scaling parameter b to the fitness analysis as they do not seem to be used in this study and are always set to 1?

This was a very helpful comment. While we think the (*a*, *b*) parameterization is defensible, we realised that our core qualitative predictions do not require these parameters at all. We have reworked our modelling approach to derive our core qualitative predictions without the need for (*a*, *b*), thus simplifying our logic. Please also see our response to Reviewer 1, comments 1-3.

11. Fig. **S2**: it looks like the lower part of panel B is cut off?

This panel appeared cut off due to a low limit of detection, leading to many missing data points for this treatment. This is not an issue anymore after repeating the experiment using *E. coli* DH5a in line with the reviewer’s other recommendations, please see revised Figure S2.
