# Supplementary material for: CRISPR-Cas is beneficial in plasmid competition, but limited by competitor toxin–antitoxin activity when horizontally transferred
Source: PLoS Biol. 2026 Feb 19;24(2):e3003658. doi: 10.1371/journal.pbio.3003658 (PMC12945316; doi:10.1371/journal.pbio.3003658)
Supplement: Attachment — Submitted filename: comments_to_reviewer.docx [file pbio.3003658.s011.docx]

Reviewer #1:

The authors have satisfactorily addressed several of my initial concerns, most notably the motivation behind the exclusion of Type IV systems and the subsequent design of the bioinformatic analysis, which I appreciate the clarification on.

However, a fundamental methodological inconsistency remains regarding the discrepancies between the model predictions and experimental results, specifically concerning the authors' defense that these discrepancies are confined to "peripheral predictions."

The current delineation between "core" and "peripheral" predictions lacks a clear, a priori justification, making the framework feel retrospective. This is particularly problematic when the primary claim established in the title is categorized in a way that allows contradictory experimental evidence to be discounted.

The first part of the title claims that "crispr-cas is beneficial in plasmid competition," which constitutes a central, core hypothesis of the manuscript. Yet, the experimental results presented in Figure 2C and 2D (specifically the square data points) clearly do not observe this beneficial effect, neither in offense nor in defense.

The diamond data points are consistent with the model predictions and corroborate the second half of the title. However, the lack of a significant effect observed with the square data points directly contradicts the first half of the title and what should be a core prediction.

Given this fundamental conflict between the primary hypothesis (as stated in the title) and the supporting data:if the claim from the title cannot be statistically assessed due to insufficient statistical power, the authors should increase the number of replicates to establish significance.

We thank the reviewer for their renewed time and comments on our manuscript. We have experimentally addressed these concerns, which directly aligns modelling outcome with experimental data across all treatments; we offer a full response below.

When conceptualising our model, our aim was to capture the outcome of plasmid competition – i.e. the relative abundance of each plasmid in different hosts, dependent on CRISPR-Cas and TA activity. The central outcome of plasmid competition forms our core model predictions. Importantly, our model agrees with our experiment when assessing qualitative variables it was designed for, i.e. the outcome of plasmid competition when changing the strength of CRISPR-Cas and/or TA. That said, we appreciate the reviewer’s concerns regarding our low limit of detection in Figure 2C, and our resulting inability to directly prove one of the model’s core predictions.

Due to experimental constraints, in the previous version of the manuscript we could not assess the plasmid content in the “CRISPR defensive” host at higher resolution: we had used DH5a::SmR for this host, which contains a chromosomal Streptomycin resistance tag. The bystander plasmid pCDF1b also encodes Streptomycin resistance, and while the bystander plasmid could be selected for in this host strain using higher Streptomycin concentrations, the bystander plasmid’s presence in both other host strains did not allow us to directly select for DH5a::SmR after mating, and to assess this strain’s plasmid content we therefore relied on picking individual colonies and stamping these onto selective media. While this method can show us broad pictures of plasmid carriage, particularly the TA-off treatments (square data points) suffered from the resulting comparatively low limit of detection, where nearly no TA plasmid RP4 was found in most samples. This was due to a strong fitness benefit of CRISPR-Cas acting as a barrier against competitor plasmid uptake. Increasing the limit of detection by picking additional colonies would be unfeasible; a power analysis indicated we would need to pick at least 4800 colonies of each of 5 biological replicates of each of the 4 treatments to give a sufficiently high limit of detection.

Therefore, we instead carried out a separate experiment using only two *E. coli* DH5a host strains. In this set up, DH5a::GmR was used as the CRISPR plasmid host (previously this was the naïve host), and DH5a::CmR was used as the TA plasmid host. Crucially, both strains have selective markers which allowed direct selective plating to assess their plasmid carriage. Additionally, streamlining this model to only two strains allows a more straightforward interpretation of results which exactly match the modelling predictions. We present the new experimental results as **Figure 2** and in lines **L190-222** (in the manuscript with tracked changes). The previous 3-strain experiment is now presented as Figure S2. We paste the new Figure 2 below. This streamlined model system has enough resolution to address the reviewer’s remaining concern.

The first part of the title claims that “CRISPR-Cas is beneficial in plasmid competition”, and both panels 2C and 2D corroborate this. Going further, we fit a GLM to our data and show that, indeed, CRISPR-Cas is always a significant determinant of the outcome of plasmid competition – both in defence and in offence **(L217-222)**

------------------------------------

Reviewer #2:

The authors have improved the manuscript through changes to the model, additional experiments and further bioinformatic analyses, addressing the reviewer comments in a satisfactory manner. The simplified model analysis and improved presentation of Figure 1 substantially increase the clarity of the model comparison and the additional bioinformatic analyses provide interesting information that lend more confidence to the results.

Thank you for your comments and time to review our manuscript.
