# Supplementary material for: CRISPR-Cas is beneficial in plasmid competition, but limited by competitor toxin–antitoxin activity when horizontally transferred
Source: PLoS Biol. 2026 Feb 19;24(2):e3003658. doi: 10.1371/journal.pbio.3003658 (PMC12945316; doi:10.1371/journal.pbio.3003658)
Supplement: Attachment — Submitted filename: addressed_comments.docx [file pbio.3003658.s012.docx]

1) Please add to your Competing Interests the following statement “SM is a member of PLOS Biology’s Editorial Board. The other authors declare that no competing interests exist.". Please add this both in your text and the metadata during resubmission.
   - Added to manuscript and metadata.
2) Please note that per journal policy, the model system/species studied (E. coli) should be clearly stated in the abstract of your manuscript.
   - Our bioinformatic analysis includes bacteria across phyla, I have reworded the abstract to include both aspects (**L29-30; L36** (Line numbers refer to tracked changes manuscript version)). I would argue that as a plasmid-focussed study, the model organism are IncP plasmids. Therefore, I have also included this in the abstract.
3) For the supplementary information that is related to methodology, could you please put in a file name "Supplementary methods" or "Mathematical model developement"? This is regarding to Suppl. results 1. Yet, we do require that you have all references in the main text, could there be a way that you can also use the citations referring  to this part in the main text?
   - All references are now included in the main text. I moved Table S1 into Methods (now Table 1), and included two additional sentences with relevant references. (**L575-6** & **L643-4**). Please note that a small number of references still exist in the supplement, but these are also included in the main text. To make this clear, I have reformatted supplementary references so there are not two separate numbering systems.

While it is ok to keep the supplementary text, as SI is so seldom read, we would recommend that Figs S1-S4 are uploaded individually with their legends, and the additional context and interpretation of the results is included in the main text, where you already describe the figure. But this will ultimately be up to you.

- Figs S1-S6 have now been uploaded individually. Additionally, I have trimmed SI (renamed to Supplementary Results) down to only include additional information for Figures S2-4. In all cases, information necessary to understand the Figures is already present in the main text. I have clarified that additional information may be found in the Supplementary Results by adding a sentence to the legend of each affected Supplementary Figure.

4) Thank you for providing the numerical values for the figures. Could you please specify to which panel in the figures the data belongs to?
   - Done, figure panels are now listed in the first spreadsheet of the supplementary excel file.
5) Please cite the location of the data clearly in all relevant main and supplementary Figure legends, e.g. “**The data underlying this Figure can be found in S1 Data**” or “The data underlying this Figure can be found in <https://doi.org/10.5281/zenodo.XXXXX>”
   - Added to all relevant Figures. (all except Figure 1)
6) Supplementary files (e.g., excel). Please ensure that all data files are uploaded as 'Supporting Information' and are invariably referred to (in the manuscript, figure legends, and the Description field when uploading your files) using the following format **verbatim**: S1 Data, S2 Data, etc. Multiple panels of a single or even several figures can be included as multiple sheets in one excel file that is saved using exactly the following convention: **S1_Data.xlsx** (using an underscore).
   - Done
7) Please ensure that your Data Statement in the submission system accurately describes where your data can be found and is in final format, as it will be published as written there
   - Done; “The data underlying this manuscript can be found in S1 Data. Code used for data gathering, processing, and visualization can be found in <https://doi.org/10.5281/zenodo.18386644> “
8) I have noticed in your acknowledgements that you thank Iolanda Domingues for input on flow cytometry. However, on the study I did not find a mention of its use. If I am mistaken, we would require the FCS files.
   - Indeed, these data were not used. To avoid confusion, I have reworded the acknowledgements to thank Iolanda Domingues for “project scoping using flow cytometry”
9) Per journal policy, if you have **generated any custom code** during the course of this investigation, please make it available without restrictions. Please ensure that the code is sufficiently well documented and reusable, and that your Data Statement in the Editorial Manager submission system accurately describes where your code can be found. More information on our Code Policy, what and how to share can be found here: <https://journals.plos.org/plosbiology/s/code-availability>
   - Done.

Additional changes

- While formatting the code for public deposition, I noticed that in the previous manuscript versions, I inadvertently copied the wrong values of a statistical model used for data presented in Figure S3A. Using the correct values has led to an additional comparison being statistically significant, and I have updated the Figure and associated Tables 6 and 7 accordingly.
